# SARS-CoV-2 Infection in Seven Childbearing Women at the Moment of Delivery, a Romanian Experience

**DOI:** 10.7759/cureus.12811

**Published:** 2021-01-20

**Authors:** Mihaela C Radu, Calin Boeru, Mihaela Marin, Loredana S Manolescu

**Affiliations:** 1 Birth, Obstetrics and Gynecology Hospital, Ploiesti, ROU; 2 Virology, Microbiology and Parasitology Discipline, University of Medicine and Pharmacy Carol Davila, Bucharest, ROU

**Keywords:** covid-19, sars-cov-2, childbearing woman, vertical transmission

## Abstract

COVID-19 pandemic outbreak had officially started on 11 March 2020 according to the World Health Organization. In Romania the first case of COVID-19 was documented on 26th of February. Ploiesti Obstetrics and Gynecology Hospital is one of the biggest mono-specialty units from Romania that was designated to deal with COVID-19 infected pregnant women. We retrospectively analyzed seven pregnant women infected with SARS-CoV-2 who gave birth during the 1st July and 30thNovember 2020. The median age of pregnancy was 39 weeks. Three of the childbearing women presented rupture membranes at hospital admission and four gave birth by cesarean section (C-section). The women infected with SARS-CoV-2 had a good evolution, vertical transmission of the virus did not occur, measures to prevent mother-to-child transmission were applied. Apgar score was 9 for all new-born babies and they all tested negative for SARS-CoV-2. There were no maternal deaths. One new born baby was preterm but didn’t present low birth weight or low Apgar score. Applying cesarean section as a method of birth did not influence vertical transmission. There is no evidence if it is necessary to anticipate the time of birth. We believe it is recommended to individualize each case according to the experience of the obstetrician and the severity of the maternal infection.

## Introduction

COVID-19 pandemic outbreak had officially started on 11 March 2020 according to the World Health Organization. Initial cases emerged in Wuhan, Hubei province, China, in December 2019. Since then the whole world has been contaminated with this viral disease. In Romania, the first case of COVID-19 was documented on 26th of February. The epidemiological reports linked it with an Italian source. In the beginning of the epidemic, there were only few cases every day, but from March 10, the number of cases increased rapidly and on 16th of March in Romania emergency state was instituted for 30 days and then prolonged for another 30 days [1].

In the early stages of the outbreak, due to the measures taken by the Romanian authorities new cases could be traced with the aid of epidemiological investigations. After the emergency state ended Romania maintained an alert state and until July 22nd the number of daily cases was below 1000. Since then, the daily number of cases increased dramatically.

At the country level there were hospitals designated as COVID-19 hospitals and here patients with the new viral disease were taken care of. There were also Hospitals of Obstetrics and Gynecology designated to deal with COVID-19 infected pregnant women. Ploiesti Obstetrics and Gynecology Hospital is one of the biggest mono-specialty units from Romania and provides specialized medical assistance (hospital and outpatient), as well as neonatal medical assistance for the inhabitants of Ploiesti county [2]. It is a category 2 obstetrics and gynecology hospital with 255 beds and 25 intensive care unit beds. The hospital is authorized to conduct clinical trials with therapeutic benefit in the clinical specialties of Obstetrics, Gynecology and Anesthesia and Intensive Care. From July 2020 the hospital was prepared to attend COVID-19 pregnant women. The staff were trained for COVID-19 case management.

Given the predominantly airborne route of transmission of SARS-CoV-2, it was essential to isolate the pregnant women confirmed or suspected of COVID-19 from the rest of non-COVID-19 patients in order to minimize the risk of contamination. Being the only maternity hospital in the county, it could not be completely transformed into a COVID-19 support hospital, so a separate section was organized for COVID-19 infected pregnant women. Circuits were organized for both hospitalization and treatment, but also for the ward of births and operating rooms. Strict isolation of COVlD-19 patients was issued with no necessary movement outside the strict indications. The transport of COVID-19 infected pregnant women throughout the hospital was performed in an organized and controlled manner (patient protected by mask, gloves, on a pre-established circuit, with staff fully equipped according to the guidelines in force and strict observance of hygiene rules, rigorous disinfection of the premises after the conclusion of the medical act of the patient with COVID-19 infection).

In pregnant women, COVID-19 increases the risk of complications such as severe pneumonia or premature birth. Acquiring the infection in the last week of pregnancy may complicate the delivery. Because the disease is highly transmissible it is essential to recognize the symptoms as quickly as possible in order to avoid transmission to new born baby. We tried to assess the outcome of delivery of the SARS-CoV-2 infected pregnant women that acquired the infection shortly before giving birth, when the virus replicates at its most.

## Materials and methods

We analyzed retrospectively seven pregnant women infected with SARS-CoV-2 who gave birth at Ploiesti Hospital during the 1st July and 30th November 2020. There was no pregnant woman excluded. These were the only childbearing women that came infected with SARS-CoV-2 for that period.

The laboratory diagnosis for COVID-19 was performed by Real-Time polymerase chain reaction (PCR) assay with manual nucleic acid extraction technique (MasterPure™ Complete DNA and RNA Purification Kit, Lucigen, Middleton, WI, USA) and RNA detection and quantification with “genesig®Real-Time PCR assay” (Primer Design™ Ltd., Camberley, UK), in vitro Real-Time PCR diagnostic test for Coronavirus (COVID-19), targeting RNA dependent RNA polymerase (RdRp) on a Real-Time PCR LightScanner 32 (LS32) (Idaho Technology, Salt Lake City, UT, USA).

## Results

The median age of the pregnant women included in the study was 28 years (limits between 23 and 43) and an average of 30.42 ± 8.12. Gestational age differed between 35 and 40 weeks with a median of 39 weeks. The hospitalization period differed by the way of birth, in case of vaginal, natural birth the women were hospitalized for four days (n = 3 women) and in case of Cesarean section (C-section) they were hospitalized for seven days (n = 4 women). There were three cases in which at the admission the childbearing women presented premature rupture of membranes.

All babies presented Apgar 9 at five minutes after birth. The average birth weight of the new born babies was 3386 ± 438 grams (no low birth weight). No baby needed the newborn intensive care unit (taking into account their COVID-19 exposure).

There was only one case in which we noticed premature birth in a pregnant woman who showed symptoms of COVID-19 infection at the time of admission and premature rupture of membranes. She gave birth at 35 weeks to a baby girl with 2900 g and Apgar 9 and stayed for seven days into the hospital. The new-born spent seven days into the hospital because this is our hospital protocol for preterm babies. No cases have been reported indicating that SARS-CoV-2 virus infection could cause developmental deficiencies in the child.

The most frequent SARS-CoV-2 symptoms encountered in the pregnant women at the time of admission were: fever (14.28%; n = 1), cough (28.57%; n = 2) and anosmia (14.28%; n = 1). The majority, 71.42% (n = 5) of the childbearing women did not present symptoms at all. These symptoms were identified at admission during the epidemiological triage. All data regarding the pregnant women and their new-born babies is presented in Table 1.

**Table 1:** Childbearing women and their newborn babies’ characteristics

Case number	1	2	3	4	5	6	7
Age (years)	24	43	23	23	33	28	39
Marital status	married	married	married	unmarried	unmarried	married	married
Provenience	urban	rural	rural	rural	urban	urban	urban
Employed	yes	no	no	no	yes	yes	yes
Number of pregnancies	1	7	2	3	1	1	2
Number of babies	1	7	2	2	1	1	1
Gestational age (weeks)	39	38	39	35	39	40	37
Symptomatic at hospital admission	No symptoms	No symptoms	No symptoms	Fever cough	No symptoms	cough anosmia	No symptoms
Delivery type	C-section	Vaginal	Vaginal	Vaginal	C-section	C-section	C-section
Rupture membranes	yes	no	yes	yes	no	no	no
Baby presentation	cephalic	cephalic	cephalic	cephalic	cephalic	cephalic	cephalic
Hospitalized (days)	7	4	4	7	7	7	7
Newborn baby’s gender	male	male	male	female	female	male	female
New born baby’s weight (grams)	3400	4100	3500	2900	3400	2800	3600
Low birth weight	no	no	no	no	no	no	no
Preterm	no	no	no	yes	no	no	no
Infant Apgar at 5 minutes	9	9	9	9	9	9	9
Infant COVID test result	negative	negative	negative	negative	negative	negative	negative
Infant location	newborn nursery	newborn nursery	newborn nursery	newborn nursery	newborn nursery	newborn nursery	newborn nursery

## Discussion

At the moment of admission in the hospital, the medical staff of the neonatology department were notified of the COVID-19 infected pregnant women’s complete status and way of delivery. The neonatologist and the neonatology nurse checked and prepared the functionality of the equipment necessary to receive the newborn at the dedicated delivery room, before the pregnant women were brought. The newborns were attended by experienced medical staff, with neonatal resuscitation skills (a primary care physician / neonatologist and a neonatology nurse). They were called at the delivery room about 15-30 minutes prior the birth procedure in order to have time to carefully equip the personal protective equipment (PPE). After birth the newborn was not placed in physical contact or at a distance of less than 2 m from the mother. The newborn babies were transported in an incubator from the delivery/operation room to the newborn nursery for isolation. All medical equipment in contact with the newborn babies from COVID-19 infected mothers was cleaned and disinfected according to disinfection protocols. During the hospitalization, the medical visits were performed by assigned specialists minimizing the contact of the number of medical staff with the patients [3].

After birth, “skin-to-skin” contact method was not performed because of increased COVID-19 infection risk to new-born from the pregnant woman, and especially because the delivery room was not provided with negative pressure.

Immediately after birth, the territorial public health and infectious diseases department was contacted to verify the case report, establish the specific treatment for COVID-19 infection and the transfer / discharge method (type of transport, destination). The follow-ups of the new mothers were performed by the obstetrician, through daily visits, with the minimum necessary duration. The medication was administered by the designated nurse. Scheduling of treatment and daily medical visits were planned so that the flow of staff in the isolation area was minimal. All the medical staff who came in contact with the new mothers wore PPE. Throughout the hospitalization, the infected COVID-19 new mothers did not leave the dedicated isolation ward.

Given the high degree of anxiety associated with the pregnancy period and the associated risk of postpartum depression, emotional support was provided during hospitalization by specialized medical staff.

In two cases, the medical condition of the new mothers didn’t allow discharge at home, so they were transferred to the infectious diseases ward because their condition worsened by the appearance of respiratory failure (saturation 91-92%).

The existing literature presents an association between preterm birth and low birth weight in the COVID-19 infected childbearing women linked by emergency cesarean section. The review conducted by Juan et al. reveals the fact that the majority of the 295 women infected with SARS-CoV-2 had delivered by cesarean section [4].

The causal relationship between SARS-CoV-2 infection and early onset of labor has not been scientifically proven. The seven childbearing women retrospectively analyzed in our study had a median age of pregnancy of 39 weeks, similar to previous studies [4], but only four women gave birth by cesarean section and neither was an emergency C-section.

Apgar score of all the new born babies was 9 and they all tested negative for SARS-CoV-2. In the literature, many studies revealed the same situation [4,5]. There were no maternal deaths. One newborn baby was preterm but didn’t present low birth weight or low Apgar score similar with Fashner and Cintron study [5].

In our study, three from seven women presented rupture membranes at the hospital admission, this is less when compared with Panahi et al.’s study where six from 29 women had premature rupture membranes [6].

For prophylaxis of COVID-19 infection the following measures, based on multidisciplinary team decisions, as in other possible viral mother-to-child transmission [7,8], were implied: artificial feeding of the new-born babies; no contact with the mothers; no delayed clamping of umbilical cord, no skin-to-skin contact; early isolation; oxygen therapy and fetal monitoring.

Intrauterine and perinatal transmission of COVID-19 infection is possible [4]. There is evidence that mother-to-child transmission is possible, although the number of publications supporting this situation is currently low.

According to national guidelines [3], the time and manner of birth for each pregnant woman with a confirmed SARS-CoV-2 infection is based on the usual obstetric indications as no benefit has been demonstrated for the caesarean section in this situation. In our study, the birth was completed by cesarean section in four cases in order to prevent the clinical deterioration of the pregnant women and to reduce the risk of fetal suffering during labor, taking into account the respiratory status of the pregnant women. It was considered that at any time the respiratory deterioration could have been appeared or worsened. In case of progressive respiratory deterioration, with the onset of respiratory failure with hypoxemia and increased need for oxygen therapy, as well as the need for ventilator support in labor, caesarean section would have been required.

The decisions to breastfeed, the care of the newborn in the rooming-in system as well as its management throughout the maternity hospital were affected by COVID-19 infection. In all cases, breastfeeding or rooming-in care system was not performed.

COVID-19 is transmitted by direct contact (less than 2 meters distance, over 15 minutes), by aerosols, by particles smaller than 5 microns, produced by laughter, loud singing and shout in the conditions of closed, unventilated spaces; so all of these were avoided.

The choice between rooming-in or mother-new born baby separation is important and the review of available cases showed that the avoidance of separation might be associated with a higher risk of late-onset neonatal SARS-CoV-2 infection. This is important since neonatal SARS-CoV-2 infections are more commonly acquired postnatal, through environmental exposure [9,10].

In our new mothers, a high level of anxiety was observed. Pregnancy is a delicate period in any woman's life when a series of hormonal, physical and mental changes occur that can increase the sensitivity of the pregnant woman. To all these factors the presence of COVID-19 infection is added and because it is a new phenomenon with limited information available, it can have negative psychological effects on pregnant women [11].

## Conclusions

The women infected with SARS-CoV-2 had a good evolution, vertical transmission of the virus did not occur, and measures to prevent vertical transmission were applied. Applying C-section as a method of birth did not influence vertical transmission. There is no evidence if it is necessary to anticipate the time of birth. We believe it is recommended to individualize each case according to the experience of the obstetrician and the severity of the maternal infection.

## References

[1] Popescu CP, Marin A, Melinte C (2020). COVID-19 in a tertiary hospital from Romania: epidemiology, preparedness and clinical challenges. Travel Med Infect Dis.

[2] Manolescu LSC, Boeru C, Căruntu C (2019). A Romanian experience of syphilis in pregnancy and childbirth. Midwifery.

[3] (2020). Methodology regarding birth in pregnancies with suspected / confirmed infection with SARS-COV-2 / COVID-19, reception, care and nursing for new born baby. https://sogr.ro/wp-content/uploads/2020/10/Actualizare-Metodologie-Octombrie-2020-Final-1.pdf.

[4] Juan J, Gil MM, Rong Z, Zhang Y, Yang H, Poon LC (2020). Effects of coronavirus disease 2019 (COVID-19) on maternal, perinatal and neonatal outcomes: a systematic review. Ultrasound Obstet Gynecol.

[5] Fashner J, Cintron C (2020). Nine SARS-CoV-2 positive pregnant women and their infant delivery outcomes. Cureus.

[6] Panahi L, Amiri M, Pouy S (2020). Risks of novel coronavirus disease (COVID-19) in pregnancy; a narrative review. Arch Acad Emerg Med.

[7] Manolescu L, Marinescu P (2013). Sex differences in HIV-1 viral load and absolute CD4 cell count in long term survivors HIV-1 infected patients from Giurgiu, Romania. Romanian Rev Lab Med.

[8] Tampa M, Mitran CI, Mitran MI (2020). The role of beta HPV types and HPV-associated inflammatory processes in cutaneous squamous cell carcinoma. J Immunol Res.

[9] Lubbe W, Botha E, Niela-Vilen H, Reimers P (2020). Breastfeeding during the COVID-19 pandemic - a literature review for clinical practice. Int Breastfeed J.

[10] Wang L, Shi Y, Xiao T (2020). Chinese expert consensus on the perinatal and neonatal management for the prevention and control of the 2019 novel coronavirus infection. Ann Transl Med.

[11] (2020). Coronavirus infection and pregnancy. https://www.rcog.org.uk/en/guidelines-research-services/guidelines/coronavirus-pregnancy/covid-19-virus-infection-and-pregnancy/.

